# Supplementary material for: Identification of photoperiod-induced specific miRNAs in the adrenal glands of Sunite sheep (Ovis aries)
Source: Front Vet Sci. 2022 Jul 22;9:888207. doi: 10.3389/fvets.2022.888207 (PMC9354845; doi:10.3389/fvets.2022.888207)
Supplement: Supplementary file 1 [file Data_Sheet_1.ZIP › Supplementary file/miRDeep2 file.zip!/miRDeep2 file/Novel_Structure/SPLP42A3/Novel_149.pdf]

Provisional ID	:	NC_019484.2_39184
Score total	:	1.9
Score for star read(s)	:	-1.3
Score for read counts	:	0
Score for mfe	:	1.6
Score for randfold	:	1.6
Score for cons. seed	:	
Total read count	:	431
Mature read count	:	431
Loop read count	:	0
Star read count	:	0

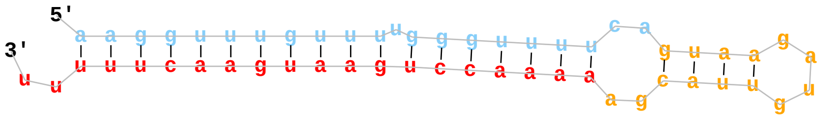

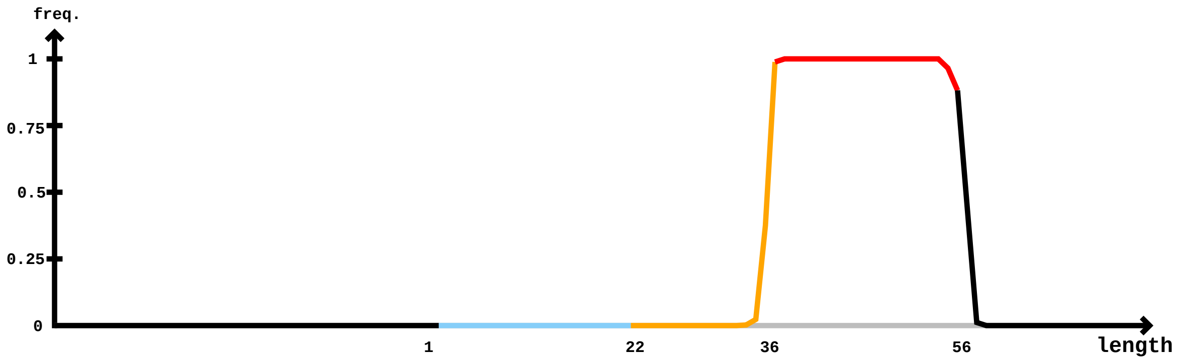

Star Mature

```
5'- acacugacugugaauauuuaaaauacuggguuggccaaaagguuuguuuuggguuuucaguaagauguuacgaaaaaccugaaugaacuuuuuguccaaccuaauauggcag   -3'   exp
    .(((.....)))..........(((.((((((((.((((((((((((((.(((((((..((((....))))..))))))))))))))))))))).)))))))).))).....   reads   mm   sample
    ......................................................................cgaaaaaccugaaugaacuuuu....................   1   0   seq
    .......................................................................gaaaaaccCgaaugaacuuu.....................   1   1   seq
    .......................................................................gaaaaaccugaaugaacuuuu....................   4   0   seq
    .......................................................................gaaaaaccAgaaugaacuuuu....................   1   1   seq
    .......................................................................gaaaaaccCgaaugaacuuuu....................   1   1   seq
    .......................................................................gGaaaaccugaaugaacuuuuu...................   1   1   seq
    .......................................................................gaaaaaccugaaCgaacuuuuu...................   1   1   seq
    ........................................................................aaaaaUcugaaugaacuuu.....................   12   1   seq
    ........................................................................aaaaaccugaaugaacCuu.....................   1   1   seq
    ........................................................................aaaaaccCgaaugaacuuu.....................   1   1   seq
    ........................................................................aaaaaUcugaaugaacuuuu....................   26   1   seq
    ........................................................................aaaaaccugaaGgaacuuuu....................   1   1   seq
    ........................................................................aaaaaUcugaaugaacuuuuu...................   78   1   seq
    ........................................................................Gaaaaccugaaugaacuuuuu...................   1   1   seq
    ........................................................................aaaaaccugaaugaacCuuu....................   1   1   seq
    ........................................................................aaaaaccugaaugaacCuuuug..................   1   1   seq
    ........................................................................aaaaaccCgaaugaacuuuuug..................   2   1   seq
    ........................................................................aaaaaccugaaCgaacuuuuug..................   3   1   seq
    ........................................................................aaaaaUcugaaugaacuuuuug..................   24   1   seq
    ........................................................................aaaaaccugaaugaacuuuuugu.................   1   0   seq
    .........................................................................aaaaccugaaugGacuuuu....................   1   1   seq
    .........................................................................aaaaccAgaaugaacuuuu....................   1   1   seq
    .........................................................................aaaCccugaaugaacuuuuu...................   1   1   seq
    .........................................................................aaaaccAgaaugaacuuuuu...................   2   1   seq
    .........................................................................aaaaccugaaugaacuuuuu...................   8   0   seq
    .........................................................................aaaaUcugaaugaacuuuuu...................   247   1   seq
    .........................................................................aaaCccugaaugaacuuuuug..................   2   1   seq
    .........................................................................aaaaccugaaugaacuuuuuU..................   2   1   seq
    .........................................................................Gaaaccugaaugaacuuuuug..................   1   1   seq
    ..........................................................................aaaccAgaaugaacuuuuug..................   1   1   seq
    ..........................................................................aaaccugaaugaacuuuuugu.................   4   0   seq
```
